# Differential Effects of Long‐Term Fertilization and Plant Species Richness on Soil Fungi and Protists

**DOI:** 10.1111/gcb.71003

**Published:** 2026-07-16

**Authors:** Peter Dietrich, Arne Schwelm, Robbert van Himbeeck, Helge Bruelheide, Stefan J. S. van de Ruitenbeek, Christiane Roscher, Stefan Geisen

**Affiliations:** ^1^ Institute of Biology/Geobotany and Botanical Garden Martin Luther University Halle‐Wittenberg Halle Germany; ^2^ German Centre of Integrative Biodiversity Research (iDiv) Halle‐Jena‐Leipzig Leipzig Germany; ^3^ Department of Environment, Soils and Landuse Teagasc Johnstown Castle Wexford Ireland; ^4^ Laboratory of Nematology, Plant Science Group Wageningen University and Research (WU) Wageningen the Netherlands; ^5^ Eukaryotic Microbiology, Faculty of Biology University of Duisburg‐Essen Essen Nordrhein‐Westfalen Germany; ^6^ Department Physiological Diversity Helmholtz Centre for Environmental Research (UFZ) Leipzig Germany

**Keywords:** biodiversity, eutrophication, global change, long read, metabarcoding, nanopore

## Abstract

Anthropogenic nutrient enrichment and plant diversity loss reshape soil biodiversity, yet disentangling their individual and combined effects on key groups such as fungi and protists remains a major challenge. Here, we investigated soil microeukaryote communities using long‐read amplicon rRNA gene sequencing in a temperate grassland experiment with 11 years of moderate NPK fertilization and manipulated plant diversity (1, 2, or 4 plant species). Our results indicate that fertilization generally had a stronger influence on microeukaryote communities than plant species richness. Fertilization altered the community composition of fungi and protists, with an increase in OTU richness by 20.8% and 52.7%, respectively, and shifted community dominance from fungi to protists. Plant diversity exclusively affected protists with a shift in community composition. Community changes were largely driven by increases in plant biomass (resulting from both fertilization and plant diversity), alongside higher soil phosphorus and lower soil pH, which were exclusively influenced by fertilization. Moreover, the experimental treatments exerted distinct effects on the different life strategies of fungi and protists. Fertilization enhanced fungal saprophytes (only richness), fungal animal pathogens, and protist consumers, whereas a decline in plant diversity increased phototrophic protists and decreased protist animal pathogens. Notably, fertilization and the decline in plant diversity together led to a cumulative increase in fungal plant pathogens. In conclusion, our results show that fertilization and reduced plant species richness exert distinct yet interacting effects on soil microeukaryotic communities. This highlights the need for integrated assessments of these two factors, rather than studying them in isolation, when evaluating global change effects.

## Graphical abstract

Fertilization strongly influences plant and soil biodiversity, yet it remains unclear whether fertilization alone drives shifts in soil biota (fungi and protists) or whether associated losses in plant diversity further intensify these effects. Moreover, it is unknown whether fertilization and plant diversity decline act through similar mechanisms or synergistically affect different soil microeukaryote groups. Our results show that fertilization has a stronger effect on soil microeukaryotic communities than plant species richness. Fertilization strongly altered fungal and protist community composition, increased OTU richness, and shifted community dominance towards protists, whereas plant diversity mainly affected protist composition. These changes were primarily linked to increases in plant biomass and fertilization‐driven changes in soil phosphorus and pH. In addition, fertilization and reduced plant diversity showed interactive effects on microbial life strategies, including cumulative increases in fungal plant pathogens. Overall, our findings demonstrate that fertilization and plant diversity exert distinct but synergistic effects on soil microeukaryotes, highlighting the need to study these global change drivers jointly rather than in isolation.

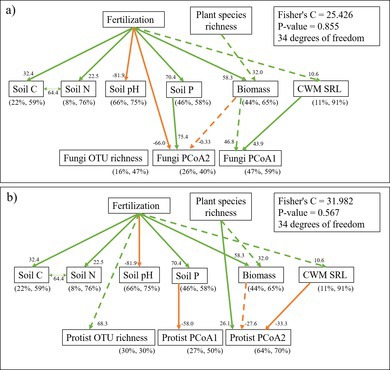

## 1 Introduction

Human activities are driving global environmental change, including climate change, biodiversity loss, and biotic homogenization. In addition to greenhouse gas emissions and land‐use change, nutrient enrichment represents a major and pervasive driver of these changes (Bobbink et al. 2010; Fowler et al. 2015; Simkin et al. 2016). For example, it has been shown that fertilization increases or decreases the soil pH depending on soil type and the type of nutrient input (Silvertown et al. 2006; Zhou et al. 2016; Ning et al. 2020). A key consequence of nutrient enrichment is the loss of plant diversity (Clark and Tilman 2008; Midolo et al. 2019; Boch et al. 2021; van der Plas et al. 2024); however, other drivers of anthropogenic plant species loss also exist, such as agricultural intensification (e.g., mowing and grazing regimes) or climate change (e.g., increased drought frequency) (Kindlund and Tyler 2023; Bakker et al. 2024). Such global change drivers often favour fast‐growing plant species characterised by acquisitive traits, such as high specific leaf area (SLA), high specific root length (SRL) and tall growth (Reich 2014; Doležal et al. 2019). These trait shifts increase competitive asymmetries, allowing dominant species to outcompete small‐statured or slow‐growing plants through shading and more efficient resource acquisition (Hautier et al. 2009). As a consequence, less competitive plant species may first decline and finally become extinct (Rajaniemi 2002; Socher et al. 2012).

Fertilization‐ and plant diversity loss‐induced changes in soil properties and plant community composition cascade through the ecosystem, altering critical processes such as decomposition and nutrient cycling by impacting soil biodiversity, including the soil fungal and protist community (Lekberg et al. 2021; Sun et al. 2024; Yang et al. 2024). In the following, we will address the specific roles of fungi and protists in soil and how they are influenced by fertilization and plant species loss.

Soil fungi play essential roles for plant community assembly and many ecosystem processes (van der Heijden et al. 2008; Bardgett and van der Putten 2014): saprotrophic fungi are key drivers of decomposition, contributing to nutrient availability, while mutualistic fungi, such as mycorrhizal species, form associations with plants and are crucial for plant nutrient uptake and growth. Conversely, the effect of pathogenic fungi can be density‐dependent, and thus, be a top‐down control mechanism promoting plant species coexistence (Wright 2002). Previous studies have shown that fertilization impacts the composition and diversity of fungal communities, with the direction of these effects varying depending on the duration and type of fertilization (Zhou et al. 2016; Wen et al. 2020; Sun et al. 2024; Yang et al. 2024), altering critical functions that soil fungi perform within ecosystems (Egerton‐Warburton et al. 2007; Liao et al. 2021). Although results remain inconsistent due to strong context dependency, overall fungal diversity may increase under fertilization. This expectation is based on the fact that a large proportion of soil fungi are saprotrophic or pathogenic, both of which have been shown to increase in abundance and diversity with fertilization (Lekberg et al. 2021; Liao et al. 2021). As these groups are closely linked to plant‐derived resources, fertilization‐driven increase in plant productivity and litter inputs are the key factors promoting fungal diversity (Liao et al. 2021). In addition, several studies have demonstrated that fertilization‐induced changes in soil properties strongly influence fungal community composition (Zhou et al. 2016; Wen et al. 2020), while more recent work highlights the important role of plant community root traits in shaping fungal communities (Hennecke et al. 2025).

With respect to plant species richness, recent studies suggest that declines in plant diversity can reduce soil fungal diversity and alter fungal functional composition (Schmid et al. 2021; Hennecke et al. 2025). The observed reduction is likely driven by decreased biomass production, litter inputs, and rhizodeposition (Hennecke et al. 2025). However, empirical evidence remains context‐dependent, with effects varying among ecosystem types and depending on environmental conditions (Maron et al. 2011; Dassen et al. 2017; Mommer et al. 2018; Schmid et al. 2021).

Protists as another important group of eukaryotic microorganisms, however, have received far less scientific attention than, e.g., fungi (Sun et al. 2024). Many protists are consumers, feeding on bacteria, which can stimulate bacterial activity, accelerate decomposition rates, and consequently increase nutrient availability for plants (Geisen et al. 2018; Hu et al. 2023). Additionally, consumers and parasitic protists can regulate the abundance of herbivores and plant pathogens (Nguyen et al. 2023). Other protists, such as plant‐pathogenic Oomycota or Plasmodiophorida also play a significant role in influencing plant community productivity (Kamoun et al. 2015; Schwelm et al. 2018). Similar to fungi, fertilization also affects the composition and diversity of protist communities (Zhao et al. 2019; Hu et al. 2024; Sun et al. 2024). Several studies demonstrated that fertilization can increase protist diversity, potentially driven by enhanced plant productivity, leading to greater litter inputs and increased bacterial abundance, which serve as a key food resource for many protists (Hu et al. 2024; Sun et al. 2024). Fertilization‐induced changes in soil properties, particularly soil pH, can also alter protist diversity and community composition, as pH strongly influences protist activity and, at least for predatory taxa, their bacterial prey (Hu et al. 2024). Whether a decline in plant species richness affects protist communities is so far unknown. There are only a few studies examining the effects of plant species richness on protists, and the reported patterns remain inconclusive (Dassen et al. 2017; Valencia et al. 2022; Solbach et al. 2026). Based on the responses observed in fungi, it can be expected that protist diversity also declines with decreasing plant diversity, likely due to reduced biomass production, associated litter inputs and reduced bacterial biomass.

In addition to the limited number of protist studies, we entirely lack insights into the combined long‐term effects of fertilization and decline of plant species richness on soil microeukaryotes. Based on current knowledge, the effects of fertilization and plant diversity loss may counterbalance each other, as the primary driver, plant biomass, is enhanced by fertilization but reduced under declining plant diversity. Consequently, the combined treatment may lead to no substantial change in fungal and protist diversity. Because plant diversity loss is a common consequence of fertilization in natural grassland systems, experimental approaches that independently manipulate nutrient inputs and plant species richness are required to disentangle these effects. Here, we studied the separate and combined effects of fertilization and plant species loss by investigating the soil fungi and protist communities in an 11‐year‐old grassland experiment differing in fertilization (either fertilized twice a year or no fertilization at all) and plant species richness (1, 2, and 4 plant species) (Siebenkäs and Roscher 2016). We took soil samples and extracted DNA for subsequent long‐read Nanopore sequencing of the rRNA operon. To explain potential effects of treatments on microeukaryote taxonomic and functional profiles, we additionally determined soil characteristics, plant biomass production, and used data on leaf and root functional traits.

We hypothesized that,

1. long‐term fertilization alters the community composition and increases the diversity of fungi and protists (a), both due to changes in soil characteristics and community‐level plant trait expression as well as increased biomass production (b);
2. a decline in plant species richness leads to shifts in the community composition and a decrease in diversity of fungi and protists, mainly due to a decline in plant biomass production;
3. fertilization and reduced plant species richness together result in antagonistic effects on fungi and protists, leading to non‐additive changes in microeukaryote community composition and diversity compared to treatments with only one of the two factors.

## 2 Materials and Methods

### 2.1 Study Site

Soil samples were collected from an experimental grassland (DivResource Experiment; Siebenkäs and Roscher (2016)), which was established in 2011 at the experimental field station of the Helmholtz Centre for Environmental Research (UFZ) in Bad Lauchstädt (Saxony‐Anhalt, Germany; 51°23′38″N, 11°52′45″E, 118 m a.s.l.). The soil is classified as Chernozem with a loamy‐sandy texture. The location had an average annual air temperature of 10.2°C and annual precipitation of 489 mm (1997–2018) (Gründling et al. 2022). Before the start of the experiment, the site was used for agricultural crop production.

Eight perennial species were selected, four herb and four grass species, which are common in mown Central European temperate grasslands (Arrhenatherion community; Table 1; Ellenberg (1988)). Two species of each functional group (grasses and herbs) were tall‐statured, whereas the other two were small‐statured. Species were randomly assigned to two species pools. Each species pool contained one tall‐statured grass and herb species and one small‐statured grass and herb species. Sown species richness levels were 1, 2 and 4 (for each species pool, respectively) with paired fertilized and unfertilized experimental plots, respectively. For both fertilization treatments (yes/no) there was one monoculture plot per species (*n* = 8 × 2), all possible two‐species combinations within the two pools (*n* = 12 × 2) and two replicates of the four‐species combination per pool (*n* = 4 × 2), resulting in a total of 48 plots (a graphical overview can be found in the Figure S1). Plots were arranged in four blocks. From 2011 to 2018, plants were grown on plots of 2 × 2 m (separated by 60 cm‐wide pathways); from 2019 onwards, plot size was reduced to 1 × 1 m by randomly choosing one quarter in each plot, which was further regularly weeded. Plant communities were mown every year in June and September. Starting in 2012, plots with fertilization were fertilized with NPK fertilizer (as pellets; 120:52:100 kg ha^−1^ year^−1^) distributed in two applications (March, June). The amount of fertilizer was moderate but equivalent to common fertilizer intensities in managed grasslands in Europe (Olff et al. 1990; Blüthgen et al. 2012) and kept the biomass production in the fertilized plots rather constant over the years, while it declined in the unfertilized plots (unpubl. results).

**TABLE 1:** Summary of plant species composition of the grassland experiment.

Species	Family	Functional group	Stature	Species pool
*Anthoxanthum odoratum* L.	Poaceae	Grass	Small	A
*Lolium perenne* L.	Poaceae	Grass	Small	B
*Arrhenatherum elatius* L. P. Beauv. ex J. Presl and C. Presl	Poaceae	Grass	Tall	A
*Dactylis glomerata* L.	Poaceae	Grass	Tall	B
*Plantago lanceolata* L.	Plantaginaceae	Herb	Small	A
*Prunella vulgaris* L.	Lamiaceae	Herb	Small	B
*Centaurea jacea* ssp. *jacea* L.	Asteraceae	Herb	Tall	A
*Knautia arvensis* (L.) Coulter	Dipsacaceae	Herb	Tall	B

*Note:* Shown are species identity, family affiliation, plant functional group affiliation, growth strategy (stature), and species pool affiliation according to Siebenkäs et al. (2017).

### 2.2 Soil Sampling

On 10 June 2022, during peak biomass season, we took six soil cores from each of the 48 plots using a 1‐cm‐diameter soil auger (each 10 cm deep). We did this in all four corners (each 30 cm from the edges of the plot) and twice in the center (50 cm from south to north edge and either 40 cm from west or east edge). Soil samples per plot were combined in a plastic bag and stored in a cool box until transported to the laboratory. Samples were sieved using a 2‐cm mesh sieve to remove stones, plant material, and larger animals. A representative subsample of the sieved soil from each plot was frozen at −20°C for later DNA analyses; the rest was stored at 4°C for soil property analyses.

### 2.3 DNA Extraction, Purification and Amplification

DNA was extracted from 1 g of soil according to Harkes et al. (2025). The DNA was subsequently used to amplify the main part of the rRNA operon (SSU‐ITS‐LSU) using the general eukaryotic primer V4F (Stoeck et al. 2010) and 21R (Schwelm et al. 2016) in duplicated PCRs. Primer pairs were barcoded with barcode sequences of the EXP‐NBD196 kit (Oxford Nanopore Technologies plc., UK) for sample multiplexing. Each PCR consisted of 12.5 μL LongAMP Taq 2× MasterMix (New England Biolabs, USA), 200 nM of each primer, 7.5 μL autoclaved Mili‐Q water and 3 ng DNA template. PCR amplification was performed with an initial step of 94°C for 90 s followed by 30 cycles of 94°C for 30 s, 59.5°C for 30 s and 65°C for 4 min 10 s and a final step of 10 min at 65°C. PCR conditions were initially based on those described by Latz et al. (2022) metabarcoding protocol and subsequently optimized using gradient PCR. Final conditions were selected based on achieving the most consistent and robust amplification. Duplicated PCRs of the same sample were pooled and the DNA concentration of the PCR products was quantified using a Qubit 3 Fluorometer. PCR negative controls did not indicate any contamination.

### 2.4 Library Preparation and Nanopore Sequencing

Sequencing libraries were generated by pooling PCR products of the samples in equimolar ratios to ensure even sequencing depth. The libraries were then cleaned using NucleoMag NGS Clean‐up and Size Select beads (0.6× bead:sample ratio) to discard unwanted artifacts and short DNA fragments (< 600 bp). Of each library, 150 fmol was prepared for nanopore sequencing using kit SQK‐LSK112 (Oxford Nanopore Technologies Plc., UK) following instructions of the manufacturer. Following library preparation, 17 fmol of each of the prepared libraries was loaded on a MinION R9.4.1 flow cell. Sequencing was performed on a MinION Mk1C (MinKNOW v22.11.2).

### 2.5 Bioinformatic Analyses

The raw nanopore reads (FAST5) were basecalled and demultiplexed using Guppy (v6.2.1, Oxford Nanopore Technologies Plc., UK) with the super‐accuracy algorithm and read‐splitting. Read splitting was enabled during basecalling to split concatenated read artefacts, a type of sequencing‐induced chimeric reads, before downstream processing. Next, the barcodes and all adapters were removed. The resulting FASTQ files were processed to polished consensus sequencing using Decona (v0.1.3) (Doorenspleet et al. 2025). Reads were filtered on quality (> Q15; NanoFilt) and length (min: 2100 bp, max 6500 bp; NanoFilt). The filtered reads were then clustered (97% identity; CD‐HIT‐EST) into OTUs and draft consensus sequences were generated (Minimap2 and Racon). Clusters containing fewer than 5 reads were removed. The draft consensus sequences were finally polished using Medaka. The resulting polished consensus sequences were identified using BLAST (Camacho et al. 2009) and the EUKARYOME v1.8 database (Tedersoo et al. 2024). Finally, we removed all OTUs that had less than 3.5 kb (the primers we used primarily amplified products larger than 3.5 kb, as demonstrated in the study by Latz et al. (2022)) as well as OTUs from multicellular organisms other than fungi and protists (i.e., metazoans and plants), due to their low number of reads. Overall, 77%, 64%, and 29% of OTUs were assigned at the family, genus, and species levels, respectively; assignment success was higher for fungi (83%, 81%, 56%) than for protists (71%, 48%, 5%). Although several hundreds of eukaryotic OTUs were detected in the whole experiment (*N* = 671), the detected richness per sample was relatively low (82.1 ± 7.1 [SE] OTUs). Nevertheless, based on the read counts (14,272.3 ± 1885.7 reads per plot; 585,165 reads in total), the main microeukaryote taxa in this experiment should be detected. Moreover, rarefaction curves approached a plateau, indicating sufficient sequencing depth (Figure S2).

### 2.6 Categorisation of OTUs Into Life Strategy Categories

Fungal OTUs were categorised into the following life strategy groups: plant pathogens, saprotrophs, microeukaryote pathogens (infesting protists/fungi), animal pathogens, and symbionts using the FungalTrait database (Põlme et al. 2020). For protists, we categorised the detected OTUs into the following groups based on Lentendu et al. (2025) and our own knowledge: plant pathogens, microeukaryote pathogens (infesting protists/fungi), animal pathogens, consumers, and phototrophic protists. The categorization aligns with the classification used in previous studies (Singer et al. 2021; Nguyen et al. 2022). OTUs were assigned to lifestyle categories based on the highest available taxonomic resolution. For fungi, 81% of OTUs were classified at least at the family level, and for protists, 71%. OTUs for which no clear assignment was possible were categorized as unclassified, accounting for 6.58% in fungi and 0.57% in protists.

For each group, the OTU richness and the percentage of reads relative to the total reads per plot were calculated. Relative OTU reads were used as a proxy for relative abundance, acknowledging known biases associated with amplicon sequencing. Hereafter, this metric is referred to as *relative abundance* throughout the manuscript.

### 2.7 Soil Properties, Plant Biomass Production and Plant Functional Traits

To test whether abiotic and biotic factors have an influence on the microeukaryote community, we measured soil pH, nitrogen, phosphorus, and potassium concentration and determined the biomass production of the plant communities. To measure soil properties, we first weighed ~10 g of fresh soil from each plot, which was then dried at 105°C for 24 h and weighed again to determine the water content and calculate the soil dry matter content. Roots and other plant material were removed from dry soil before grinding a subsample into a fine powder using a mixer mill (MM2000, Retsch, Germany). The concentrations of total nitrogen (N) and total carbon (C) were subsequently analyzed using an elemental analyzer (Vario EL cube, Elementar Analysensysteme GmbH, Germany). For the measurement of pH, potassium, and phosphorus, we used fresh soil stored in the fridge (4°C for a maximum of 4 weeks). Soil pH was determined in a soil‐water suspension using a calibrated pH meter (WTW inoLab ph 7110, Xylem Analytics, Germany). Soil potassium (K) was assessed by percolation with a 0.2 N BaCl_2_ solution, followed by measurement using atomic absorption spectrometry (AAS Vario 6, Analytik Jena, Germany). Plant‐available phosphorus was extracted using the double lactate method (2 g fresh soil) and measured photometrically using the molybdate‐ascorbic acid method (blue colouration).

To assess aboveground biomass, plants were harvested at a height of three centimetres above the ground in a 20 × 50 cm area per plot (random position leaving out the outer 20 cm of the plot area) in late May 2022. Plant biomass was sorted into plant functional groups (grasses and forbs) and dead material. After drying the samples for 48 h at 70°C, dry biomass of sown species was weighed and extrapolated to one square‐metre.

Functional traits of plant species, i.e., specific leaf area (SLA) and specific root length (SRL) were obtained from Siebenkäs et al. (2016). These traits were selected because they capture key axes of plant resource acquisition strategies above‐ and belowground and are closely linked to plant–soil interactions and nutrient cycling. Using these values, we calculated community‐weighted means (CWMs) per plot. These are mean trait values weighted by species relative abundances according to the equation:

$${\mathrm{CMW}}_{\mathrm{SLA}/\mathrm{SRL}}=\sum_{i=1}^{S}p_{i}t_{i}$$

where *S* is the number of species in the community, *p*_*i*_ are the species biomass proportions, and *t*_*i*_ are species‐specific SLA or SRL values. For calculation, biomass data from 2023 were used because species‐level biomass sorting was conducted in that year. Comparative analyses based on plant species cover data showed that species' relative abundances remained stable between 2022 and 2023.

### 2.8 Statistical Analyses

To test the hypotheses how fertilization (yes/no), plant species richness (1, 2, 4 species, as numeric predictor), and their interaction influence the microeukaryote community, we employed multivariate analysis to assess differences in community composition as well as linear mixed‐effects models to examine differences in the abundance and diversity of fungi and protists. Multivariate analysis included principal coordinate analysis (PCoA) and permutational multivariate analysis of variance (PERMANOVA), using Bray–Curtis dissimilarity both for the fungal and protist communities. Bray–Curtis dissimilarities were calculated using OTU relative abundances. With PERMANOVA, we tested the effect of plant species richness, fertilization and their interaction (plant species richness × fertilization) on the community composition both of fungi and protists using 999 permutations.

For linear mixed‐effects models, we started with a null model with the random effects block and plant mixture identity (i.e., plant species combination) only, and then extended the model stepwise by adding plant species richness, fertilization, and the interaction (plant species richness × fertilization) as fixed effects. Response variables are shown in Table 2. We excluded the following life strategy groups from the analysis, as these groups showed too low OTU richness/relative abundance: fungal symbionts, microeukaryote pathogens (protists) and plant pathogens (protists). Mixed‐effects models were fitted with maximum likelihood (ML), and likelihood ratio tests were used to compare models and assess the significance of the fixed effects.

**TABLE 2:** Summary of mixed‐effects model analyses testing the effects of plant species richness, fertilization and their interaction on OTU richness and relative abundance of the microeukaryote community.

Taxonomic and functional groups	OTU richness									Relative abundance								
	Plant species richness			Fertilization			Plant species richness × fertilization			Plant species richness			Fertilization			Plant species richness × fertilization		
	Chi^2^	*p*	Ef.	Chi^2^	*p*	Ef.	Chi^2^	*p*	Ef.	Chi^2^	*p*	Ef.	Chi^2^	*p*	Ef.	Chi^2^	*p*	Ef.
Total microeukaryote community	0.40	0.525		7.22	**0.007**	**↑**	0.55	0.457		—	—		—	—		—	—	
Fungi:Protist ratio	0.95	0.329		8.90	**0.003**	**↓**	0.89	0.344		< 0.01	0.937		10.14	**0.002**	**↓**	< 0.01	0.963	
Fungal community	0.10	0.748		5.30	**0.021**	**↑**	0.20	0.658		< 0.01	0.950		10.57	**0.001**	**↓**	< 0.01	0.938	
Ascomycota (46%)	0.08	0.777		5.45	**0.020**	**↑**	0.05	0.817		0.09	0.343		1.94	0.163		1.47	0.225	
Basidiomycota (28%)	< 0.01	0.980		2.13	0.145		1.85	0.174		1.67	0.196		4.60	**0.032**	**↓**	2.70	0.100	
Non‐Dikarya (26%)	1.35	0.246		7.24	**0.007**	**↑**	0.03	0.854		0.56	0.456		0.56	0.456		0.99	0.320	
Protist community	0.81	0.368		9.64	**0.002**	**↑**	0.57	0.451		< 0.01	0.950		10.57	**0.001**	**↑**	< 0.01	0.938	
Alveolata (17%)	3.68	*0.055*	**↑**	2.89	*0.089*	**↑**	< 0.01	0.948		10.05	**0.002**	**↑**	0.28	0.594		0.48	0.487	
Amoebozoa (15%)	0.65	0.421		0.55	0.456		1.28	0.258		2.62	0.106		10.40	**0.001**	**↓**	2.00	0.158	
Rhizaria (49%)	1.29	0.257		15.64	**< 0.001**	**↑**	0.79	0.375		0.08	0.776		36.07	**< 0.001**	**↑**	0.14	0.706	
Stramenopiles (7%)	0.88	0.348		< 0.01	0.955		2.27	0.132		2.36	0.125		5.10	**0.024**	**↓**	0.66	0.418	
Chlorophyta (8%)	7.00	**0.008**	**↓**	0.14	0.704		0.44	0.506		5.10	**0.024**	**↓**	0.37	0.541		0.45	0.502	
Fungal life strategies																		
Animal pathogens (5%)	< 0.01	0.958		7.77	**0.005**	**↑**	0.61	0.435		0.13	0.719		9.50	**0.002**	**↑**	1.97	0.160	
Microeukaryote pathogens (9%)	< 0.01	0.937		2.47	0.116		0.20	0.655		0.09	0.766		< 0.01	0.972		4.23	**0.040**	**↓:↑**
Plant pathogens (11%)	1.07	0.302		10.27	**0.001**	**↑**	0.18	0.665		4.80	**0.028**	**↓**	4.95	**0.026**	**↑**	0.56	0.453	
Saprophytes (66%)	0.06	0.811		3.86	**0.050**	**↑**	0.52	0.472		0.80	0.370		16.57	**< 0.001**	**↓**	0.15	0.696	
Protist life strategies																		
Animal pathogens (7%)	6.40	**0.011**	**↑**	2.26	0.133		0.05	0.833		12.36	**< 0.001**	**↑**	0.17	0.682		1.69	0.193	
Consumer (78%)	0.78	0.379		11.88	**< 0.001**	**↑**	0.33	0.568		1.93	0.164		13.10	**< 0.001**	**↑**	1.29	0.256	
Phototrophic protists (11%)	5.83	**0.016**	**↓**	< 0.01	0.929		1.14	0.285		5.89	**0.015**	**↓**	1.39	0.238		0.53	0.467	

*Note:* Shown are Chi‐square, *p*‐values (values with *p* < 0.05 are shown in bold; values with 0.05 < *p* < 0.10 are shown in italics) and effect direction (Ef.), i.e., positive effects are indicated by an upward‐pointing arrow, negative effects by a downward‐pointing arrow, significant interaction effects by two arrows (↓:↑). The percentage values for taxonomic and functional groups represent the average proportion of OTUs relative to the total number of OTUs. Note that degrees of freedom (DF) was one for all fixed effects fitted in the models.

To test how soil and plant variables influence the microeukaryote community, we first did a principal component analysis (PCA) with soil properties (N, P, and pH), plant biomass production, and CWMs of SLA and SRL (soil K was excluded due to its strong correlation with soil P, and soil C due to its strong correlation with soil N). In a second step, we tested the effect of these variables on OTU richness and relative abundance, using the same mixed‐effects model approach as described above, with block and plant mixture identity as random effects, and in separate models, PC scores (PC1–3), soil properties, plant biomass, or plant traits as fixed effects. We also carried out PERMANOVAs with PC scores, soil properties, plant biomass production, or plant traits.

Moreover, to check for direct and indirect effects of plant and soil variables on eukaryote diversity and community composition, we applied piecewise structural equation modelling (SEM). We started with an initial SEM that included fertilization (0 = no, 1 = yes) and plant species richness as exogenous variables. These factors were modelled as first‐order drivers of soil N, soil C, soil P, soil pH, plant biomass, and the CWM of specific root length (SRL). In addition, the model included OTU richness and the first two ordination axes (PCoA1 and PCoA2) derived from the PCoA described above as response variables. Separate SEMs were fitted for fungal and protist communities. SEMs were based on mixed effects models accounting for block and plant mixture identity as random effects, as it was done in all previous mixed‐effects models. Model fit was assessed using Fisher's C statistic, where *p* > 0.05 indicates that the data are well represented by the model.

Prior to all analyses, variables were transformed to meet the assumptions of normality and variance homogeneity. All analyses were performed with the statistical software R (version 3.6.1, R Development Core Team, http://www.R‐project.org). For community composition analysis, we used capscale, anova and adonis2 functions of the R package vegan (Oksanen et al. 2007), for linear mixed‐effects models, we used the lmer function in the R package lme4 (Bates et al. 2015); for PCA the fviz_pca_biplot function of the R packages FactoMineR (Lê et al. 2008) and factoextra (Kassambara and Mundt 2017) as well as the package ggplot2 (Wickham et al. 2016), and for SEMs we employed the psem function of the R package piecewiseSEM (Lefcheck 2016).

## 3 Results

We detected a total of 670 microeukaryotic OTUs, comprising 318 fungal OTUs (47.5%), and 352 protist OTUs (52.5%). On average, we detected 82.1 ± 7.1 (SE) OTUs per plot with 14272.3 ± 1885.7 reads. Fungal communities were dominated by Ascomycota and Basidiomycota, whereas protist communities were primarily composed of Rhizaria, followed by Amoebozoa, Alveolata, and Chlorophyta. Across life strategies, fungi were largely saprotrophic, while protists were dominated by consumer taxa, with pathogens representing a smaller but consistent fraction in both groups.

### 3.1 Influence of Fertilization and Plant Species Richness on the Community Composition, Relative Abundance and Richness of Fungi and Protists

**FIGURE 1:**
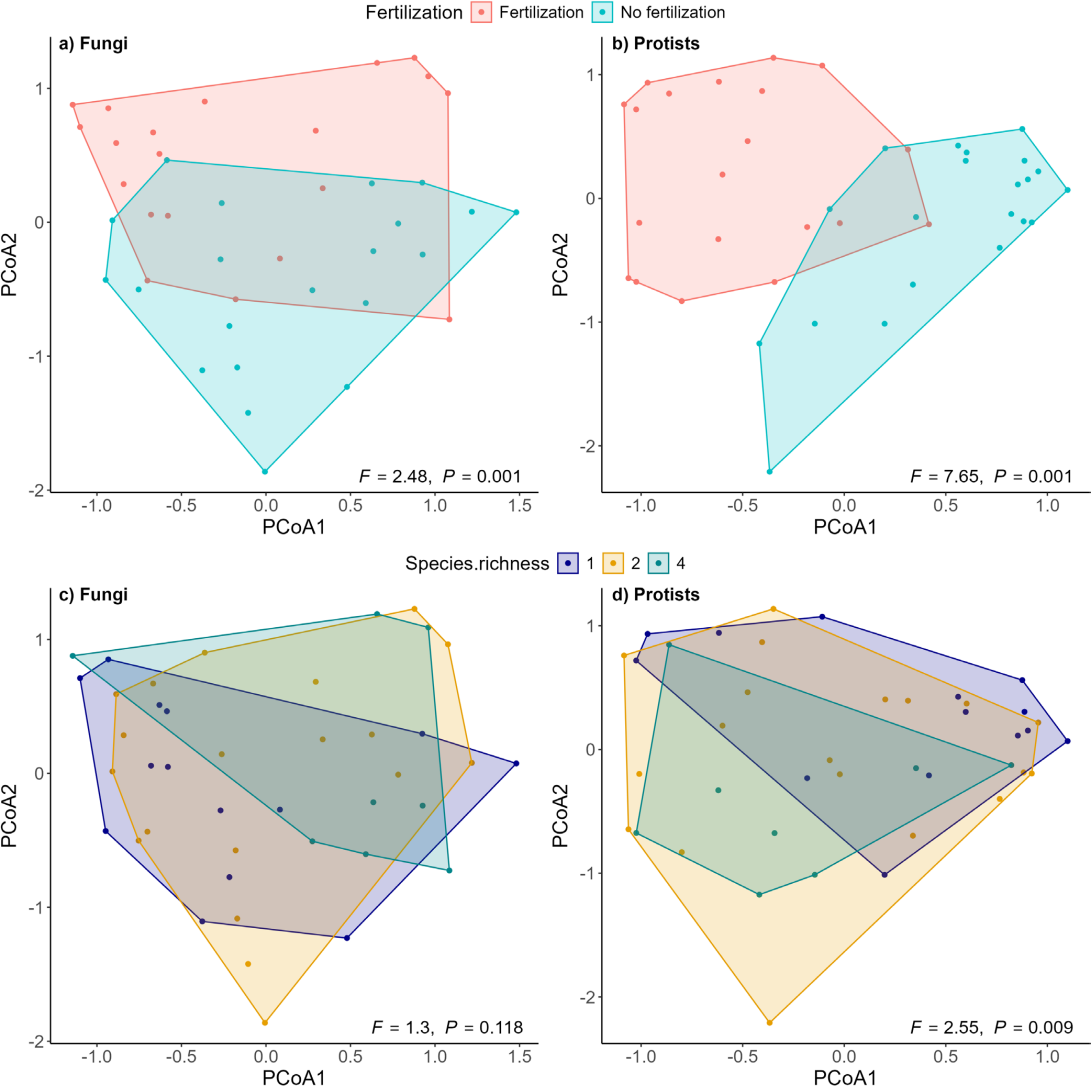
First two axes of principal coordinates analysis (PCoA), based on the community composition data (relative abundances on OTU level) of fungi (a, c) and protists (b, d), shown by treatments, either fertilization (a, b) or plant species richness (c, d). Points represent research plots.

Fertilization influenced the community composition of fungi (*F*_1,37_ = 2.48, *p* = 0.001) and protists (*F*_1,37_ = 7.65, *p* = 0.001), while plant species richness only affected protist (*F*_1,37_ = 2.55, *p* = 0.009) but not fungal community composition (*F*_1,37_ = 1.30, *p* = 0.118; Figure 1). Interactions between fertilization and plant species richness did impact neither bacterial nor fungal community compositions (fungi: *F*_1,37_ = 0.67, *p* = 0.936; protists: *F*_1,37_ = 0.71, *p* = 0.782).

Fertilization increased total OTU richness by 25.3 OTUs on average (+36.2%; Table 2). Moreover, fertilization decreased the ratio between fungal and protist OTU abundance and the ratio between fungal and protist OTU richness, indicating a shift from a dominance of fungi (mean F:P ratio reads: 1.48; mean F:P ratio richness: 1.13) to a protist‐dominated system (mean F:P ratio reads: 0.90; mean F:P ratio richness: 0.89; Table 2). There was no plant species richness or interaction effect on total OTU richness or F:P ratios (Table 2).

Among fungi, there was a significant effect of fertilization on both OTU richness and relative abundance (Table 2). Fungal OTU richness increased with fertilization (+7.5 OTUs avg.), mainly due to an increase in OTU richness of Ascomycota (+5 OTUs avg.) and Non‐Dikarya (+1.3 OTUs avg.; Table 2). Fungal relative abundance decreased with fertilization (−10.3% on average [avg.]), which is mainly explainable by a decrease in relative abundance of Basidiomycota (−5.4% avg.; Table 2). Plant species richness and its interaction with fertilization did neither influence fungal OTU richness nor relative abundance (Table 2).

We also identified a strong influence of fertilization on the protist community: Protist OTU richness increased with fertilization (+17.8 OTUs avg.), due to an increase in Alveolata (+1.1 OTUs avg.) and Rhizaria (+16 OTUs avg.; Table 2). Protist relative abundance overall increased with fertilization (+10.3% avg.), due to an increase in relative abundance of Rhizaria (+19.6% avg.); although, we also detected a significant decrease in relative abundance of Amoebozoa (−7.5% avg.) and Stramenopiles (−0.8% avg.; Table 2). Plant species richness increased Alveolata OTU richness (+2.4 OTUs avg.) and relative abundance (+19% avg. from 1 to 4 plant species), and reduced Chlorophyta OTU richness (−3.2 OTUs avg.) and relative abundance (−7% avg.; Table 2).

### 3.2 Influence of Fertilization and Plant Species Richness on Life Strategies of Fungi and Protists

**FIGURE 2:**
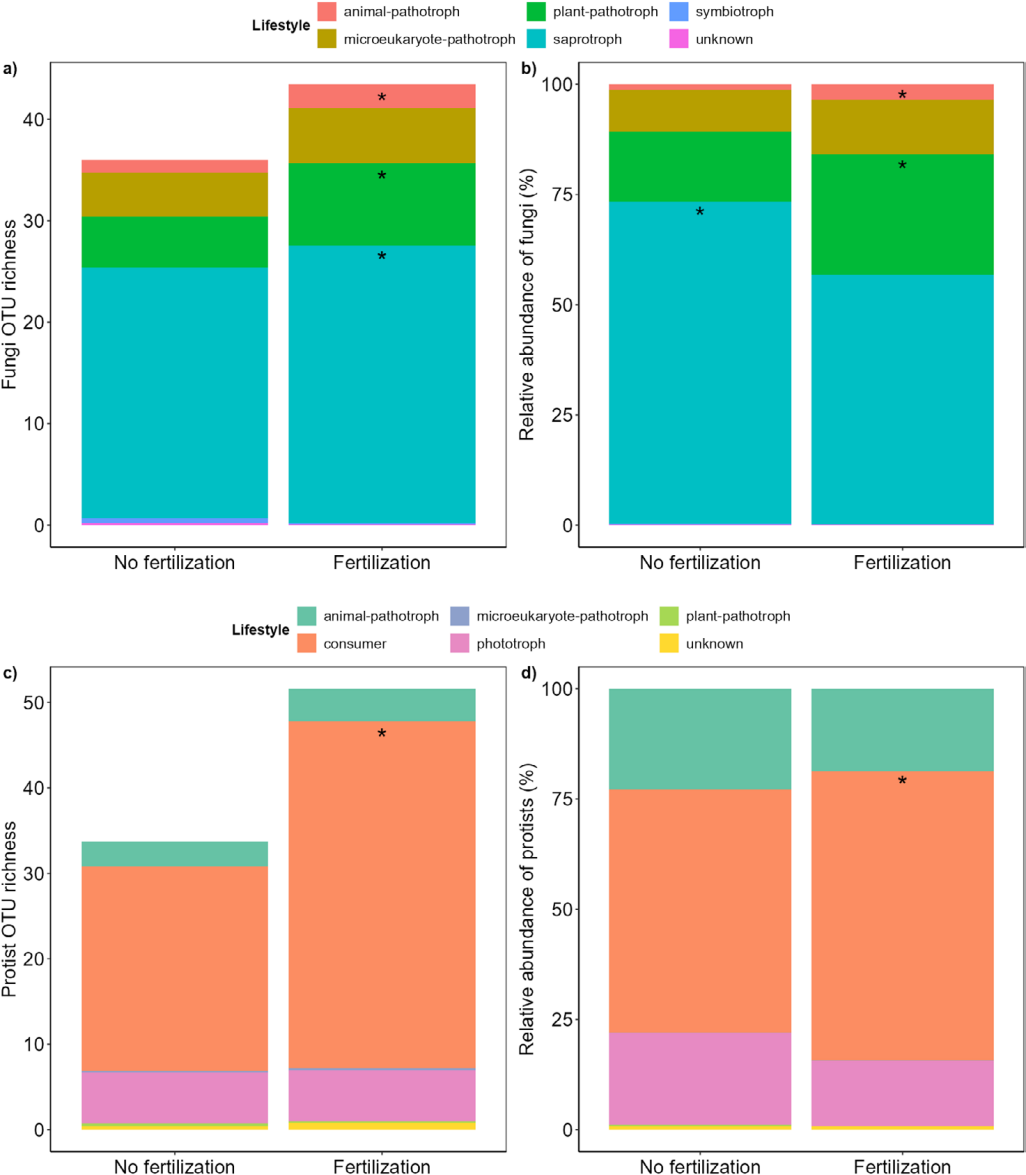
OTU richness and relative abundance of fungal (a, b) and protist (c, d) life strategy groups in soil of unfertilized and fertilized plant communities. Asterisks within the bars indicate significant differences (*p* < 0.05) related to life strategy groups (the value was significantly higher at the treatment marked with the asterisk).

Fertilization increased the OTU richness and relative abundance of fungal plant pathogens (+3 OTUs, +2.0% avg.), fungal animal pathogens (+1.1 OTUs, +2.3% avg.), and protist consumers (+16.6 OTUs, +12.1% avg.; Table 2 and Figure 2). In the case of fungal saprophytes, fertilization increased OTU richness (+2.6 OTUs avg.), but decreased relative abundance (−13.6% avg.; Table 2 and Figure 2). Relative abundance of microeukaryote pathogens (fungi) also decreased with fertilization, but only in four‐plant species communities, which resulted in a significant interaction between fertilization and plant richness (4: −8.7% avg.; Table 2).

**FIGURE 3:**
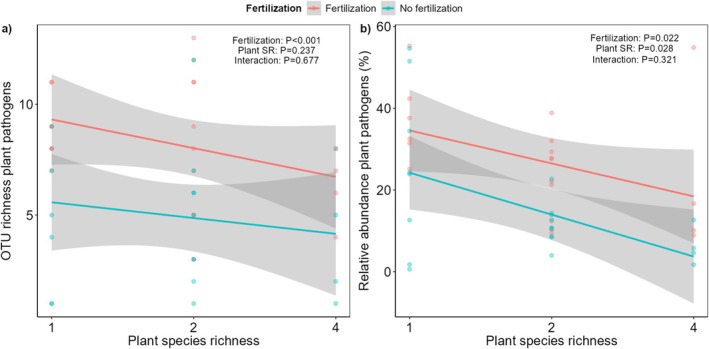
Effects of fertilization and plant species richness on fungal plant pathogens. Shown is OTU richness of plant pathogens (a) and relative abundance of plant pathogens (b) in response to plant species richness (log) under fertilized (red) and non‐fertilized (blue) conditions. Shaded areas represent 95% confidence intervals. Statistical results from linear models testing for the effects of fertilization, plant species richness (Plant SR), and their interaction are provided in the panels.

Higher plant species richness caused lower relative abundance of fungal plant pathogens (−9.5% avg. from 1 to 4 plant species; Figure 3). Moreover, plant species richness decreased relative abundance and richness of phototrophic protists (−7.8%, −3.8 OTUs avg.), as well as enhanced protist relative abundance and richness of animal pathogens (+34.7%, +1.9 OTUs avg.; Table 2). When analysing effects on total plant pathogens, microeukaryote pathogens and animal pathogens (i.e., fungi + protists), the same patterns were obtained as described above.

### 3.3 Mediating Effects of Fertilization and Plant Species Richness

Principal component analysis (PCA) revealed that PC1 was negatively associated with soil pH and positively with soil phosphorus (P) and plant biomass, while PC2 was positively linked to plant traits (high values of SLA and SRL), and PC3 to soil nitrogen conentration (mean (±SD) values of plant biomass and soil properties across monocultures, 2‐, and 4‐species mixtures under fertilized and unfertilized conditions are presented in Table S1; PCA can be found in Figure S3). Fertilization significantly influenced PC1 (DF = 1, Chi^2^ = 55.45, *p* < 0.001) and PC2 (DF = 1, Chi^2^ = 17.10, P < 0.001), indicating an increase in soil P, plant biomass, and CWM of SRL and SLA, as well as a decrease in soil pH with fertilization (Figure S3). Plant species richness and their interaction had no effect on PC axes. PC3 was not affected by any of the factors.

**FIGURE 4:**
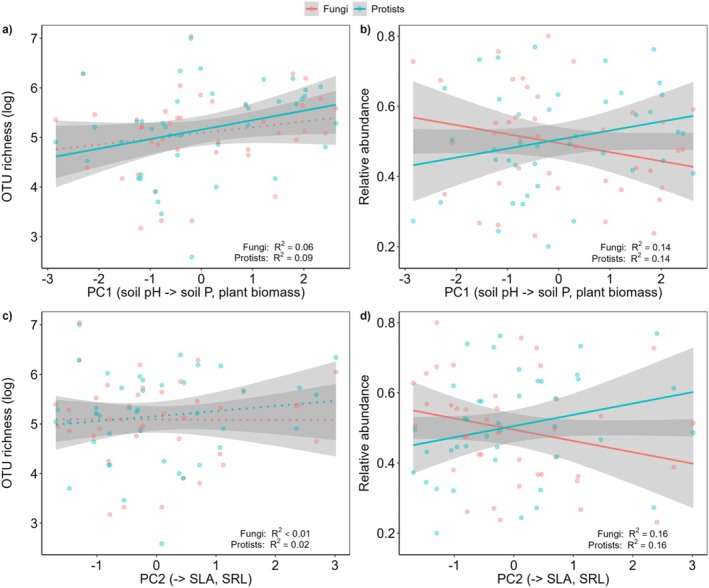
Relationships between microeukaryotes and soil and plant variables. Panels (a, b) show the effects of principal component 1 (PC1; low scores for high values of soil pH, high scores for high values of soil phosphorous and plant biomass) on (a) OTU richness (log‐transformed) and (b) relative abundance. Panels (c, d) show the effects of principal component 2 (PC2; high scores for high community‐level SLA and SRL) on (c) OTU richness (log‐transformed) and (d) relative abundance. Red lines represent fungi and blue lines represent protists; shaded areas indicate 95% confidence intervals, and points represent individual observations. Solid lines indicate significant relationships, whereas dotted lines indicate non‐significant relationships. Regression lines are based on linear models, with corresponding marginal *R* ^2^ values shown in each panel.

PERMANOVA showed that fungal and protist community composition was mainly influenced by soil P, plant biomass and soil pH (PC1; fungi: *F* = 2.25, *p* = 0.002; protist: *F* = 8.66, *p* = 0.001), while fungal community composition was additionally influenced by plant traits (PC2; *F* = 1.81, *p* = 0.004; Table S2). Mixed‐effects model analysis showed that soil P and plant biomass (PC1) generally had a positive influence on OTU richness (i.e., total OTU richness of protists, OTU richness of various groups), whereas soil pH had a negative effect (PC1; Table 3 and Figure 4). Other factors, such as plant traits (PC2) and soil N (PC3) only influenced OTU richness of a few groups (Table 3 and Figure 4). Regarding relative abundances, soil P, plant biomass, SLA, and SRL (PC1 and PC2) had a positive influence on protists, particularly consumers, while pH (PC1) negatively affected them (Table 3 and Figure 4). The opposite pattern was observed for fungi, especially saprophytes (Table 3 and Figure 4). Soil nitrogen (PC3) negatively affected the relative abundances of animal pathogens but positively influenced consumers (Table 3). Results on single effects of plant and soil variables can be found in the supplement (Tables S3 and S4).

**TABLE 3:** Summary of mixed‐effects model analyses testing the effects of the leading principal components (PC1; low scores for high values of soil pH, high scores for high values of soil phosphorous and plant biomass), PC2 (high scores for high community‐level SLA and SRL) and PC3 (high scores for high soil nitrogen) on OTU richness and relative abundance of the microeukaryote community.

	OTU richness									Relative abundance								
	PC1 (pH‐> P, biomass)			PC2 (‐> SLA, SRL)			PC3 (‐> N)			PC1 (pH‐> P, biomass)			PC2 (‐> SLA, SRL)			PC3 (‐> N)		
	Chi^2^	*p*	Ef.	Chi^2^	*p*	Ef.	Chi^2^	*p*	Ef.	Chi^2^	*p*	Ef.	Chi^2^	*p*	Ef.	Chi^2^	*p*	Ef.
Total microeuk. community	3.08	*0.079*	↑	0.22	0.636		1.68	0.195		—	—		—	—		—	—	
F:P ratio	4.43	**0.035**	↓	4.09	**0.043**	↓	2.84	*0.092*	↓	8.54	**0.003**	↓	5.54	**0.019**	↓	0.34	0.558	
Fungal community	0.36	0.548		0.01	0.913		0.36	0.548		8.93	**0.003**	↓	5.81	**0.016**	↓	0.37	0.543	
Protist community	3.94	**0.047**	↑	0.75	0.388		2.54	0.111		8.93	**0.003**	↑	5.81	**0.016**	↑	0.37	0.543	
Fungal life strategies																		
Animal pathogens (5%)	5.12	**0.024**	↑	0.88	0.349		0.39	0.535		8.10	**0.004**	↑	1.85	0.174		0.06	0.815	
Microeukaryote pathogens (9%)	1.38	0.240		0.19	0.663		1.99	0.158		0.02	0.891		0.07	0.794		3.09	*0.079*	↓
Plant pathogens (11%)	5.55	**0.019**	↑	1.10	0.294		0.04	0.835		1.66	0.198		2.33	0.127		0.13	0.714	
Saprophytes (66%)	0.13	0.714		1.54	0.214		< 0.01	0.931		11.94	**< 0.001**	↓	11.19	**< 0.001**	↓	0.01	0.906	
Protist life strategies																		
Animal pathogens (7%)	4.93	**0.026**	↑	2.06	0.152		0.59	0.443		0.38	0.535		0.84	0.358		7.08	**0.008**	↓
Consumer (78%)	4.77	**0.029**	↑	1.10	0.293		1.86	0.170		7.04	**0.008**	↑	3.54	*0.056*	↑	4.20	**0.040**	↑
Phototrophic protists (11%)	0.36	0.547		0.03	0.856		3.97	**0.046**	↑	3.24	*0.072*	↓	1.20	0.274		0.32	0.573	

*Note:* Shown are Chi‐square, *p*‐values (values with *p* < 0.05 are shown in bold; values with 0.05 < *p* < 0.10 are shown in italics) and effect direction (Ef.), i.e., positive effects are indicated by an upward‐pointing arrow, negative effects by a downward‐pointing arrow. The percentage values for fungal and protist life strategies represent the average proportion of OTUs relative to the total number of OTUs in the respective groups. Note that degrees of freedom (DF) was one for all fixed effects fitted in the models.

**FIGURE 5:**
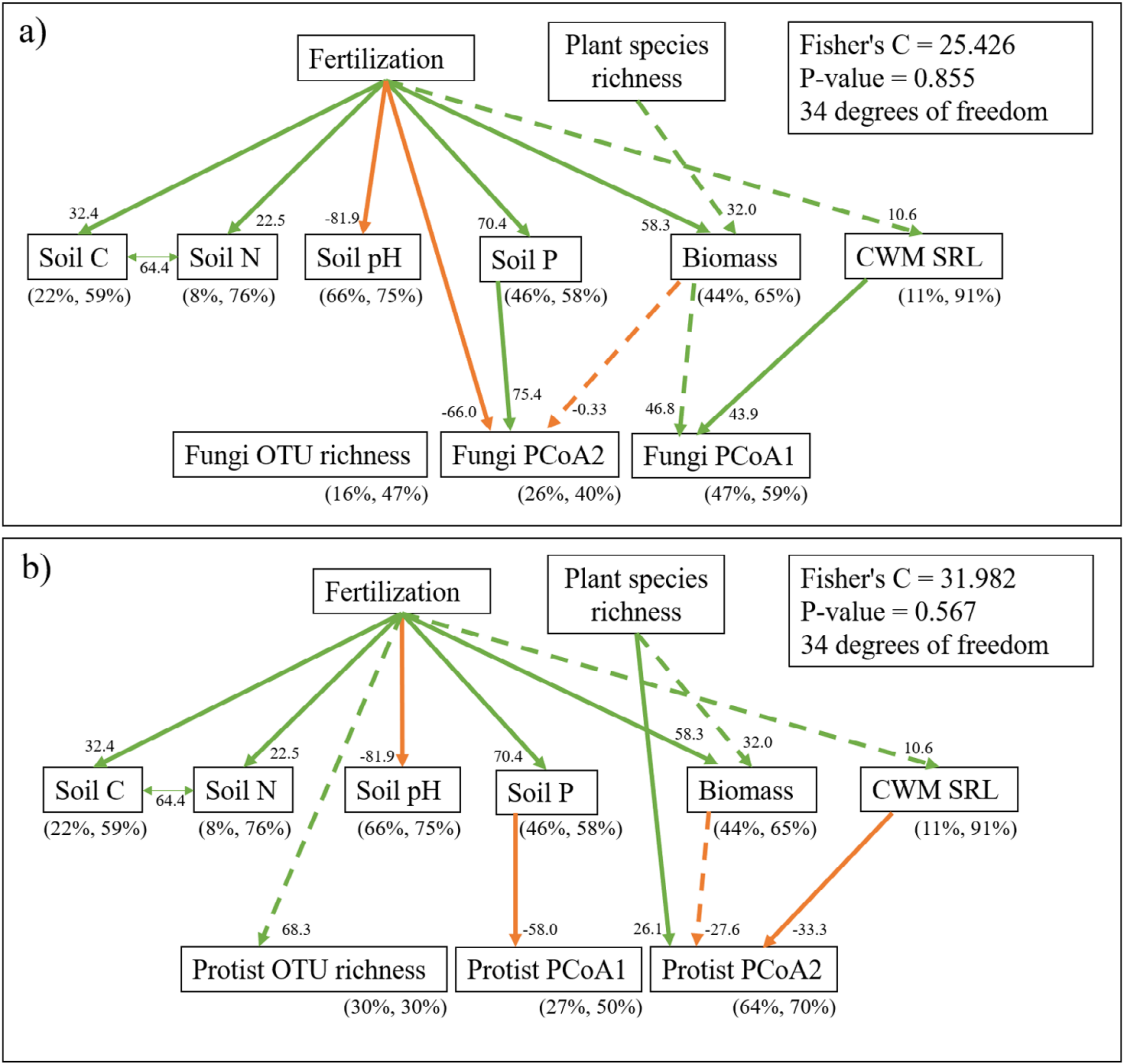
Piecewise structural equation model (SEM) examining the effects of fertilization, plant species richness, soil characteristics, and plant traits (specific root length [SRL]) and biomass on OTU richness and community composition (PCoA axes 1 and 2) of fungi (a) and protists (b). Arrows represent significant unidirectional relationships among variables (*p* < 0.05). Green arrows indicate positive relationships, whereas red arrows indicate negative relationships. Marginally significant relationships are indicated by dashed arrows (*p* ≤ 0.065) for visualization purposes. Non‐significant relationships are not shown. Standardized parameter estimates are shown next to the arrows. Marginal *R* ^2^ (variance explained by fixed effects only) and conditional *R* ^2^ (variance explained by fixed and random effects) for component models with significant relationships are shown in brackets below the respective response variables.

The SEM analyses yielded largely consistent results. Fertilization generally increased soil nutrient concentrations, soil carbon, plant biomass, and CWM of specific root length (SRL), while reducing soil pH (Figure 5). In addition, plant species richness had a positive effect on plant biomass (Figure 5). Fungal OTU richness was unaffected by fertilization or plant species richness (Figure 5a), whereas protist OTU richness showed a marginally significant positive response to fertilization (Figure 5b). With respect to community composition, fertilization exerted a direct effect on fungal PCoA1, together with additional indirect pathways associated with soil phosphorus, biomass, and the CWM of specific root length (SRL; Figure 5a). Protist community composition was similarly associated with soil phosphorus, biomass, and SRL and additionally showed a direct effect of plant species richness on PCoA2 (Figure 5b).

## 4 Discussion

### 4.1 (H1a) Fertilization Shifted the Community Composition and Increased Diversity of Fungi and Protists

The community composition of fungi and protists was shaped by fertilization. Other studies also demonstrated the strong influence of fertilization on the composition of soil fungi (Zhou et al. 2016; Wen et al. 2020) and soil protists (Zhao et al. 2019; Hu et al. 2024; Sun et al. 2024). In addition, protists appear to be more sensitive to fertilization than fungi (Zhao et al. 2019; Sun et al. 2024), which we confirm in our study.

Fertilization also affected the richness and relative abundance of fungi and protists. We found that fertilization generally enhanced the OTU richness of fungi and even more of protists. As most protists are predominantly bacterivores (Geisen et al. 2018), and that fertilization especially promotes bacteria over fungi (de Vries et al. 2006; Hu et al. 2024), a shift towards the bacterial‐dominated system can be assumed. An increase in fungal and protist richness is in line with findings from previous studies (Zhou et al. 2016; Zhao et al. 2019). However, other studies have reported a decrease in richness (Wen et al. 2020; Hu et al. 2024), suggesting that the direction of the fertilization effect likely depends on multiple factors, such as the type of fertilization, soil characteristics, and plant community composition (Ullah et al. 2019; Ye et al. 2020). Notably, the majority of long‐term fertilization studies on fungi and protists so far have been conducted in China (Zhou et al. 2016; Ullah et al. 2019; Zhao et al. 2019; Wen et al. 2020; Hu et al. 2024; Sun et al. 2024). Our results confirm similar patterns for a European temperate grassland site, thereby expanding current knowledge beyond the predominantly studied Chinese systems and providing evidence that similar processes may also operate in European temperate grasslands.

### 4.2 (H1b) Fertilization Shifted the Fungi and Protist Community by Modifying Soil Properties and Plant Biomass Production

Fertilization can influence plant communities through two primary pathways: increased nutrient availability, which enhances biomass production, as well as soil acidification (Maskell et al. 2010). In our study, these processes also emerged as key drivers of changes in microeukaryotes, which were primarily shaped by plant biomass, soil pH and soil phosphorus.

In general, increased plant biomass had a positive effect on fungal and protist diversity (with the exception of Chlorophyta), likely due to enhanced resource availability (e.g., prey or hosts), consistent with findings by Sun et al. (2024). A primary explanation for the increased abundance of fungal plant pathogens under fertilization is the higher biomass production, as greater biomass increases the root surface area available for pathogen colonization. Fertilization was further associated with higher abundances of consumer protists, potentially driven by increased bacterial biomass resulting from greater litter inputs linked to higher plant productivity (total soil carbon increased from 2.8% to 3.0% in fertilized plots, suggesting higher litter input and/or increased microbial biomass). In addition, the observed increase in animal pathogens may reflect larger arthropod populations supported by increased plant biomass (Prather et al. 2020).

Another plant‐related pathway beyond biomass enhancement involves fertilization‐induced shifts in plant functional traits toward higher specific root length (SRL) and specific leaf area (SLA). In our experiment, this shift is likely driven by an increasing dominance of fast‐growing grasses, outcompeting slower‐growing forbs under fertilization (You et al. 2017; Nelson et al. 2025). Consistent with a recent study showing that high SRL values favour fungal pathogens while reducing saprotrophic fungi (Hennecke et al. 2025), we observed similar patterns in our data. Moreover, our results demonstrate that fertilization‐driven shifts toward a faster plant resource‐use strategy also restructure protist community composition and functional groups.

Moreover, our results indicate that fertilization‐induced soil acidification promotes protist relative abundance and richness, but limits fungi. The observed shift in the fungi‐to‐protist ratio with fertilization may be explained by differing pH growth optima or tolerance ranges among fungi, protists, and the bacteria that serve as protist prey (Ye et al. 2020). Previous studies have demonstrated a significant impact of fertilizer‐induced pH changes on fungi (Zhou et al. 2016; Ullah et al. 2019; Ning et al. 2020; Ye et al. 2020), and to a lesser extent on protists (Ye et al. 2020).

Beyond biomass, plant traits and pH, soil phosphorus concentration (P) emerges as another key driver of community composition and diversity. Increased soil phosphorus availability can alter fungal and protist communities by relaxing nutrient limitation and favoring taxa adapted to resource‐rich conditions. In fungi, higher P availability may reduce the importance of nutrient‐acquisition strategies, leading to shifts towards faster‐growing taxa. In protists, soil phosphorus primarily influences community composition indirectly by altering the abundance and quality of bacterial prey, driven by shifts towards faster‐growing metabolic strategies (Fierer et al. 2007).

### 4.3 (H2) Plant Species Richness Shifted the Community Composition of Protists and Diversity of Specific Groups of Protists and Fungi

In general, we observed fewer effects of plant species richness on protists and fungi than those of fertilization, which is in line with the results from other studies that manipulated plant richness (Waldrop et al. 2006; Dassen et al. 2017; Mommer et al. 2018, but also see Schmid et al. (2021) for fungal diversity). In terms of fungi, the only group significantly affected by plant species richness was that of plant pathogens, which increased in relative abundance from 4‐species communities to monocultures. Numerous studies propose that the decline in plant productivity within species‐poor communities may result from the accumulation of plant pathogens, while in diverse plant mixtures, a dilution effect of competent hosts (Keesing and Ostfeld 2021) results in more stable productivity (Maron et al. 2011; Schnitzer et al. 2011; Mommer et al. 2018). However, evidence of pathogen accumulation in biodiversity experiments has only been documented in a few studies so far (Mommer et al. 2018; van Ruijven et al. 2020; Wang et al. 2023; Hennecke et al. 2025). Our findings provide further support that increased plant diversity can reduce pathogen loads.

Related to protists, we found that plant species richness affected community composition, as well as richness and abundance of phototrophic protists (Chlorophyta) and animal pathogens. Differences in composition can be explained by variations in bacterial communities driven by plant diversity (Schmid et al. 2021), given that a significant proportion of the protist community consisted of (mostly bacterivorous) consumers (66%). Additionally, the observed increase in animal pathogens and the concurrent decrease in phototrophic protists (Chlorophyta) may partially contribute to the variations in protist community composition associated with plant species richness. The SEM indicated that the effects of plant species richness are partly mediated by plant biomass. Higher biomass production in species‐rich mixtures may account for the increased abundance of animal pathogens and the reduced abundance of phototrophic protists, likely through increased host availability and reduced light penetration resulting from greater plant biomass. Here, we show that a further reduction in species richness in already species‐poor plant communities can strongly influence protist communities. This affects not only overall community composition but also selectively promotes or suppresses specific protist groups, consistent with recent findings by Solbach et al. (2026) across a broader species‐richness gradient.

### 4.4 (H3) No Antagonistic Interaction Effects of Fertilization and Plant Diversity on Fungi or Protists

We observed no antagonistic effects of fertilization and plant species richness, largely because the two drivers influenced distinct microeukaryotic groups. Fertilization primarily increased the OTU richness of fungal plant and animal pathogens, saprophytes, and protist consumers, whereas plant species richness mainly affected phototrophic protists and protist animal pathogens. This highlights that fertilization and reductions in plant species richness independently shape different components of soil microeukaryotic communities. When these drivers act simultaneously, as it is common in managed ecosystems (Socher et al. 2012), their combined effects can therefore result in more pronounced and comprehensive shifts in soil community composition than either factor acting alone. Together, these findings highlight the importance of considering multiple, co‐occurring global change drivers to understand and predict belowground biodiversity responses.

Nevertheless, we observed one important cumulative effect: both fertilization and reduced plant species richness increased the relative abundance of plant pathogens. This highlights the importance of pathogen dilution mediated by plant diversity (Wang et al. 2023), as its loss facilitates pathogen transmission among neighbouring plants and promotes pathogen accumulation. Fertilization further amplifies this effect by potentially promoting fungal spore production, increasing infection success, and facilitating lesion growth by plant hosts (Veresoglou et al. 2013; Liu et al. 2016). In managed ecosystems, increased nutrient input can promote plant pathogens, potentially triggering cascading effects in which particularly poor competitors are lost under combined nutrient enrichment and elevated pathogen pressure (Liu et al. 2017). Such losses may further weaken the dilution effect, reinforcing pathogen dominance and accelerating plant decline. Our results indicate that fertilization therefore has not only immediate effects on soil communities but also persistent consequences by undermining biotic regulatory mechanisms such as disease dilution. These findings emphasise that reducing nutrient pollution alone is unlikely to be sufficient for soil ecosystem recovery; restoring diverse plant communities is essential to re‐establish soil biodiversity and associated ecosystem functions.

Finally, we would like to point out that our study was based on well‐controlled gradients, with an overall low plant species richness representing three levels (1, 2, and 4 species) and fertilization applied at moderate intensity. This particular focus of our experiment should be considered when interpreting the generality of the observed diversity and fertilization effects. At the same time, this approach enabled us to isolate and assess global change effects under conditions that reflect realistic management scenarios. Within this framework, our results show that halving the species number in plant communities with already low plant species richness (e.g., from four to two species), together with nutrient inputs, which basically replaced the amount of nutrients which were annually removed with the mown biomass (unpubl. results) can substantially influence micro‐eukaryotic diversity and community composition.

## 5 Conclusion

By disentangling the effects of fertilization and reduced plant species richness, our study demonstrates that these two drivers exert distinct influences on soil microeukaryotic communities. While fertilization directly modifies soil conditions and plant biomass production, loss in plant species richness and changes in community trait values represent an additional and independent pathway through which belowground communities are affected. This insight may help explain the variability in reported fertilization effects across studies in which plant diversity dynamics were not explicitly accounted for. Moreover, we show that even moderate, long‐term fertilization combined with a further reduction in plant species richness in plant communities with already low plant species richness can lead to pronounced shifts in soil microeukaryotic communities. Together, these findings highlight the importance of long‐term experiments that allow direct fertilization effects to be separated from indirect effects mediated by plant species decline, while focusing on experimentally realistic levels of nutrient enrichment and biodiversity change representative of managed grassland ecosystems.

## Author Contributions

**Peter Dietrich:** conceptualization, investigation, writing – original draft, methodology, formal analysis, visualization. **Stefan Geisen:** funding acquisition, writing – review and editing, supervision, resources, conceptualization. **Christiane Roscher:** resources, writing – review and editing, project administration. **Stefan J. S. van de Ruitenbeek:** data curation, methodology, writing – review and editing. **Arne Schwelm:** methodology, writing – review and editing. **Helge Bruelheide:** writing – review and editing, resources, supervision. **Robbert van Himbeeck:** data curation, methodology, writing – review and editing.

## Funding

This work was supported by German Academic Exchange Service.

## Conflicts of Interest

The authors declare no conflicts of interest.

## Supporting information


**Table S1:** Mean (± SD) of plant biomass and soil properties under different levels of plant diversity and fertilization. Values are shown for monocultures, 2‐species mixtures, and 4‐species mixtures under fertilized and unfertilized conditions. Soil properties include phosphorus (P), potassium (K), nitrogen (N), carbon (C), and pH.
**Table S2:** Summary of PERMANOVA testing the effects of soil PC1, PC2 and PC3 on community composition of fungi and protists. Shown are model output of the likelihood ratio tests, i.e., Chi^2^‐ and *p*‐values, and Coefficient of determination (*R* ^2^).
**Table S3:** Summary of mixed‐effects model analyses testing the effects of plant and soil characteristics on OTU richness and relative abundance of the microeukaryote community. Shown are model outputs from likelihood ratio tests, including degrees of freedom (DF), Chi‐square, *p*‐values and Coefficient of determination (*R* ^2^). Significant positive effects (*p* < 0.05) are indicated by an upward‐pointing arrow, and significant negative effects by a downward‐pointing arrow.
**Table S4:** Summary of mixed‐effects model analyses testing the effects of plant and soil characteristics on OTU richness and relative abundance of the microeukaryote community. Shown are model outputs from likelihood ratio tests, including degrees of freedom (DF), Chi‐square, *p*‐values and Coefficient of determination (*R* ^2^). Significant positive effects (*p* < 0.05) are indicated by an upward‐pointing arrow, and significant negative effects by a downward‐pointing arrow.
**Figure S1:** Overview of the experimental design of the Grassland Experiment (DivResource) for one species pool (= 24 plots × 2 species pools = 48 plots in total).
**Figure S2:** Rarefaction curves showing observed microeukaryotic richness as a function of sequencing depth across all samples.
**Figure S3:** Multivariate ordination of soil properties, plant traits (SLA, SRL), and plant biomass in relation to fertilization treatment. (a) Principal component analysis (PCA) showing separation of samples along PC1 and PC2. (b) PCA showing separation along PC1 and PC3. Points represent individual plots, and coloured ellipses indicate 95% confidence intervals for fertilization treatments (red = no fertilization, blue = fertilization). Arrows represent environmental and plant trait vectors, with arrow length and direction indicating the strength and direction of correlations with ordination axes. Percentages on axes indicate the proportion of variance explained by each dimension.

## Data Availability

The nanopore sequencing data of the soil microeukaryote communities are publicly available at BioStudies under number E‐MTAB‐15101 (https://www.ebi.ac.uk/biostudies/arrayexpress/studies/E‐MTAB‐15101). All additional data supporting this study are openly available on Zenodo under https://doi.org/10.5281/zenodo.21216635.
