# Supplementary material for: Differential Effects of Long‐Term Fertilization and Plant Species Richness on Soil Fungi and Protists
Source: Glob Chang Biol. 2026 Jul 16;32(7):e71003. doi: 10.1111/gcb.71003 (PMC13375590; doi:10.1111/gcb.71003)
Supplement: Supplementary file 1 — Table S1: Mean (± SD) of plant biomass and soil properties under different levels of plant diversity and fertilization. Values are shown for monocultures, 2‐species mixtures, and 4‐species mixtures under fertilized and unfertilized conditions. Soil properties include phosphorus (P), potassium (K), nitrogen (N), carbon (C), and pH. Table S2: Summary of PERMANOVA testing the effects of soil PC1, PC2 and PC3 on community composition of fungi and protists. Shown are model output of the likelihood ratio tests, i.e., Chi2‐ and p‐values, and Coefficient of determination (R 2). Table S3: Summary of mixed‐effects model analyses testing the effects of plant and soil characteristics on OTU richness and relative abundance of the microeukaryote community. Shown are model outputs from likelihood ratio tests, including degrees of freedom (DF), Chi‐square, p‐values and Coefficient of determination (R 2). Significant positive effects (p < 0.05) are indicated by an upward‐pointing arrow, and significant negative effects by a downward‐pointing arrow. Table S4: Summary of mixed‐effects model analyses testing the effects of plant and soil characteristics on OTU richness and relative abundance of the microeukaryote community. Shown are model outputs from likelihood ratio tests, including degrees of freedom (DF), Chi‐square, p‐values and Coefficient of determination (R 2). Significant positive effects (p < 0.05) are indicated by an upward‐pointing arrow, and significant negative effects by a downward‐pointing arrow. Figure S1: Overview of the experimental design of the Grassland Experiment (DivResource) for one species pool (= 24 plots × 2 species pools = 48 plots in total). Figure S2: Rarefaction curves showing observed microeukaryotic richness as a function of sequencing depth across all samples. Figure S3: Multivariate ordination of soil properties, plant traits (SLA, SRL), and plant biomass in relation to fertilization treatment. (a) Principal component analysis (PCA) showing separati [file GCB-32-e71003-s001.docx]

**Supplement**

**Differential effects of long-term fertilization and plant species richness on soil fungi and** **protists** by Peter Dietrich, Arne Schwelm, Robbert van Himbeeck, Helge Bruelheide, Stefan van de Ruitenbeek, Christiane Roscher, and Stefan Geisen

**Table S1** Mean (± SD) of plant biomass and soil properties under different levels of plant diversity and fertilization. Values are shown for monocultures, 2-species mixtures, and 4-species mixtures under fertilized and unfertilized conditions. Soil properties include phosphorus (P), potassium (K), nitrogen (N), carbon (C), and pH.

|  | Plant biomass  [g/m^2^] | Soil P [ug/g] | Soil K [ug/g] | Soil N [%] | Soil C [%] | Soil pH |
| --- | --- | --- | --- | --- | --- | --- |
| Without fertilization |  |  |  |  |  |  |
| Monocultures | 131.2 (±90.6) | 100.6 (±40.6) | 247.6 (±95.2) | 0.20 (±0.02) | 2.68 (±0.22) | 6.93 (±0.18) |
| 2-species mixtures | 135.3 (±76.2) | 79.7 (±15.1) | 333.3 (±151.1) | 0.22 (±0.03) | 2.91 (±0.28) | 6.80 (±0.21) |
| 4-species mixtures | 173.0 (±47.4) | 133.4 (±20.4) | 283.0 (±51.9) | 0.21 (±0.02) | 2.93 (±0.21) | 6.80 (±0.07) |
| With fertilization |  |  |  |  |  |  |
| Monocultures | 258.0 (±194.9) | 133.4 (±20.4) | 354.4 (±43.0) | 0.22 (±0.03) | 2.87 (±0.22) | 6.05 (±0.16) |
| 2-species mixtures | 371.8 (±151.3) | 143.6 (±12.7) | 364.9 (±110.9) | 0.23 (±0.03) | 3.00 (±0.20) | 6.15 (±0.33) |
| 4-species mixtures | 540.5 (±82.3) | 135.1 (±22.2) | 367.3 (±28.3) | 0.24 (±0.03) | 3.07 (±0.05) | 6.06 (±0.35) |

**Table S2** Summary of PERMANOVA testing the effects of soil PC1, PC2 and PC3 on community composition of fungi and protists. Shown are model output of the likelihood ratio tests, i.e., Chi^2^- and P-values, and Coefficient of determination (R^2^).

|  | Fungi | | | Protists | | |
| --- | --- | --- | --- | --- | --- | --- |
|  | F | P | R^2^ | F | P | R^2^ |
| PC1 (pH->soil P, plant biomass) | 2.25 | **0.002** | 0.055 | 8.66 | **0.001** | 0.182 |
| PC2 (->SLA, SRL) | 1.81 | **0.004** | 0.044 | 1.30 | 0.296 | 0.032 |
| PC3 (-> soil N) | 1.39 | *0.061* | 0.035 | 1.20 | 0.254 | 0.030 |

**Table S3** Summary of mixed-effects model analyses testing the effects of plant and soil characteristics on OTU richness and relative abundance of the microeukaryote community. Shown are model outputs from likelihood ratio tests, including degrees of freedom (DF), Chi-square, P-values and Coefficient of determination (R^2^). Significant positive effects (P < 0.05) are indicated by an upward-pointing arrow, and significant negative effects by a downward-pointing arrow.

|  | Total OTU richness | | |  | | |
| --- | --- | --- | --- | --- | --- | --- |
|  | Chi^2^ | P | Effect & R^2^ |  |  |  |
| Plant biomass | 3.81 | *0.051* | ↑ 0.097 |  |  |  |
| Specific root length | 1.04 | 0.307 |  |  |  |  |
| Specific leaf area | 0.60 | 0.438 |  |  |  |  |
| Soil nitrogen | 1.60 | 0.205 |  |  |  |  |
| Soil phosphorus | 0.08 | 0.773 |  |  |  |  |
| Soil potassium | 1.31 | 0.252 |  |  |  |  |
| Soil pH | 3.88 | **0.049** | ↓ 0.092 |  |  |  |
|  | F:P ratio richness | | | F:P ratio relative abundance | | |
|  | Chi^2^ | P | Effect & R^2^ | Chi^2^ | P | Effect & R^2^ |
| Plant biomass | 0.73 | 0.390 |  | 0.14 | 0.710 |  |
| Specific root length | 3.03 | *0.082* | ↓ 0.079 | 0.10 | 0.753 |  |
| Specific leaf area | 0.40 | 0.526 |  | 2.33 | 0.127 |  |
| Soil nitrogen | 4.17 | **0.041** | ↓ 0.112 | 2.33 | 0.127 |  |
| Soil phosphorus | 3.99 | **0.046** | ↓ 0.087 | 10.86 | **<0.001** | ↓ 0.166 |
| Soil potassium | 0.24 | 0.627 |  | 0.91 | 0.340 |  |
| Soil pH | 5.56 | **0.018** | ↑ 0.114 | 8.71 | **0.003** | ↑ 0.139 |
|  | Fungi OTU richness | | | Fungi OTU relative abundance | | |
|  | Chi^2^ | P | Effect & R^2^ | Chi^2^ | P | Effect & R^2^ |
| Plant biomass | 4.22 | **0.040** | ↑ 0.100 | 0.16 | 0.689 |  |
| Specific root length | 0.41 | 0.521 |  | 0.14 | 0.710 |  |
| Specific leaf area | 1.07 | 0.300 |  | 2.28 | 0.131 |  |
| Soil nitrogen | 0.41 | 0.523 |  | 2.51 | 0.113 |  |
| Soil phosphorus | 0.08 | 0.778 |  | 11.79 | **<0.001** | ↓ 0.170 |
| Soil potassium | 1.20 | 0.273 |  | 0.95 | 0.330 |  |
| Soil pH | 2.28 | 0.131 |  | 8.81 | **0.003** | ↑ 0.137 |
|  | Protist OTU richness | | | Protist OTU relative abundance | | |
|  | Chi^2^ | P | Effect & R^2^ | Chi^2^ | P | Effect & R^2^ |
| Plant biomass | 3.65 | *0.056* | ↑ 0.087 | 0.16 | 0.689 |  |
| Specific root length | 1.81 | 0.179 |  | 0.14 | 0.710 |  |
| Specific leaf area | 0.30 | 0.586 |  | 2.28 | 0.131 |  |
| Soil nitrogen | 2.52 | 0.112 |  | 2.51 | 0.113 |  |
| Soil phosphorus | 0.40 | 0.528 |  | 11.79 | **<0.001** | ↑ 0.170 |
| Soil potassium | 0.87 | 0.350 |  | 0.95 | 0.330 |  |
| Soil pH | 5.09 | **0.024** | ↓ 0.119 | 8.81 | **0.003** | ↓ 0.137 |

**Table S4** Summary of mixed-effects model analyses testing the effects of plant and soil characteristics on OTU richness and relative abundance of the microeukaryote community. Shown are model outputs from likelihood ratio tests, including degrees of freedom (DF), Chi-square, P-values and Coefficient of determination (R^2^). Significant positive effects (P < 0.05) are indicated by an upward-pointing arrow, and significant negative effects by a downward-pointing arrow.

|  | Plant pathogen richness (fungi) | | | Plant pathogen relative abundance (fungi) | | |
| --- | --- | --- | --- | --- | --- | --- |
|  | Chi^2^ | P | Effect & R^2^ | Chi^2^ | P | Effect & R^2^ |
| Plant biomass | 7.42 | **0.006** | ↑ 0.185 | 5.72 | **0.017** | ↑ 0.105 |
| Specific root length | 2.79 | *0.095* | ↑ 0.067 | 9.80 | **0.002** | ↑ 0.265 |
| Specific leaf area | 1.23 | 0.267 |  | 0.71 | 0.401 |  |
| Soil nitrogen | 0.10 | 0.752 |  | 0.01 | 0.929 |  |
| Soil phosphorus | 2.88 | *0.090* | ↑ 0.085 | 0.60 | 0.438 |  |
| Soil potassium | 0.57 | 0.452 |  | 0.76 | 0.382 |  |
| Soil pH | 4.38 | **0.036** | ↓ 0.109 | 1.68 | 0.195 |  |
|  | Saprophyte richness (fungi) | | | Saprophyte relative abundance (fungi) | | |
|  | Chi^2^ | P | Effect & R^2^ | Chi^2^ | P | Effect & R^2^ |
| Plant biomass | 3.16 | *0.076* | ↑ 0.068 | 1.87 | 0.171 |  |
| Specific root length | 0.22 | 0.636 |  | 3.19 | *0.074* | ↓ 0.087 |
| Specific leaf area | 0.79 | 0.374 |  | 2.50 | 0.114 |  |
| Soil nitrogen | 0.69 | 0.405 |  | 0.37 | 0.544 |  |
| Soil phosphorus | <0.01 | 0.983 |  | 12.69 | **<0.001** | ↓ 0.224 |
| Soil potassium | 2.30 | 0.129 |  | 1.43 | 0.232 |  |
| Soil pH | 1.33 | 0.249 |  | 13.20 | **<0.001** | ↑ 0.207 |
|  | Microeukaryote path. richness (fungi) | | | Microeukaryote path. relative abundance (f.) | | |
|  | Chi^2^ | P | Effect & R^2^ | Chi^2^ | P | Effect & R^2^ |
| Plant biomass | 0.57 | 0.450 |  | 0.10 | 0.749 |  |
| Specific root length | 0.03 | 0.862 |  | 0.22 | 0.642 |  |
| Specific leaf area | 0.59 | 0.442 |  | <0.01 | 0.980 |  |
| Soil nitrogen | 1.06 | 0.302 |  | 2.91 | *0.088* | ↓ 0.069 |
| Soil phosphorus | 1.04 | 0.308 |  | <0.01 | 0.926 |  |
| Soil potassium | 0.58 | 0.446 |  | 1.42 | 0.234 |  |
| Soil pH | 2.78 | *0.095* | ↓ 0.057 | 0.49 | 0.482 |  |
|  | Animal pathogen richness (fungi) | | | Animal pathogen relative abundance (fungi) | | |
|  | Chi^2^ | P | Effect & R^2^ | Chi^2^ | P | Effect & R^2^ |
| Plant biomass | 2.17 | 0.141 |  | 2.11 | 0.146 |  |
| Specific root length | 1.02 | 0.312 |  | 0.29 | 0.591 |  |
| Specific leaf area | 0.56 | 0.453 |  | 0.10 | 0.755 |  |
| Soil nitrogen | 1.49 | 0.221 |  | 0.60 | 0.440 |  |
| Soil phosphorus | 5.29 | **0.021** | ↑ 0.068 | 11.75 | **<0.001** | ↑ 0.254 |
| Soil potassium | 2.16 | 0.142 |  | 10.51 | **0.001** | ↑ 0.230 |
| Soil pH | 3.62 | *0.057* | ↓ 0.086 | 6.40 | **0.011** | ↓ 0.148 |
|  | Consumer richness (protist) | | | Consumer relative abundance (protist) | | |
|  | Chi^2^ | P | Effect & R^2^ | Chi^2^ | P | Effect & R^2^ |
| Plant biomass | 5.14 | **0.023** | ↑ 0.120 | 3.84 | **0.050** | ↑ 0.129 |
| Specific root length | 1.84 | 0.175 |  | 2.98 | *0.084* | ↑ 0.072 |
| Specific leaf area | 0.18 | 0.675 |  | 0.61 | 0.436 |  |
| Soil nitrogen | 1.74 | 0.187 |  | 4.30 | **0.038** | ↑ 0.108 |
| Soil phosphorus | 0.35 | 0.553 |  | 1.39 | 0.239 |  |
| Soil potassium | 1.21 | 0.272 |  | 1.04 | 0.308 |  |
| Soil pH | 7.10 | **0.008** | ↓ 0.162 | 7.88 | **0.005** | ↓ 0.185 |
|  | Phototrophic protists richness (protist) | | | Phototrophic protists abundance (protist) | | |
|  | Chi^2^ | P | Effect & R^2^ | Chi^2^ | P | Effect & R^2^ |
| Plant biomass | 0.28 | 0.594 |  | 8.63 | **0.003** | ↓ 0.112 |
| Specific root length | 2.8 | *0.094* | ↑ 0.080 | 2.59 | 0.107 |  |
| Specific leaf area | 0.69 | 0.406 |  | 1.27 | 0.259 |  |
| Soil nitrogen | 1.48 | 0.223 |  | 0.15 | 0.695 |  |
| Soil phosphorus | 1.10 | 0.295 |  | 0.37 | 0.543 |  |
| Soil potassium | 2.56 | 0.110 |  | 0.10 | 0.750 |  |
| Soil pH | 0.01 | 0.911 |  | 0.42 | 0.519 |  |
|  | Animal pathogen richness (protist) | | | Animal pathogen relative abundance (protist) | | |
|  | Chi^2^ | P | Effect & R^2^ | Chi^2^ | P | Effect & R^2^ |
| Plant biomass | 3.65 | *0.056* | ↑ 0.087 | 0.57 | 0.449 |  |
| Specific root length | 2.29 | 0.130 |  | 9.86 | **0.002** | ↓ 0.271 |
| Specific leaf area | 1.80 | 0.179 |  | 0.04 | 0.846 |  |
| Soil nitrogen | 4.45 | **0.035** | ↑ 0.105 | 1.37 | 0.242 |  |
| Soil phosphorus | 2.50 | 0.114 |  | 3.11 | *0.078* | ↑ 0.049 |
| Soil potassium | 0.35 | 0.552 |  | 4.78 | **0.029** | ↑ 0.082 |
| Soil pH | 0.24 | 0.625 |  | 0.01 | 0.912 |  |


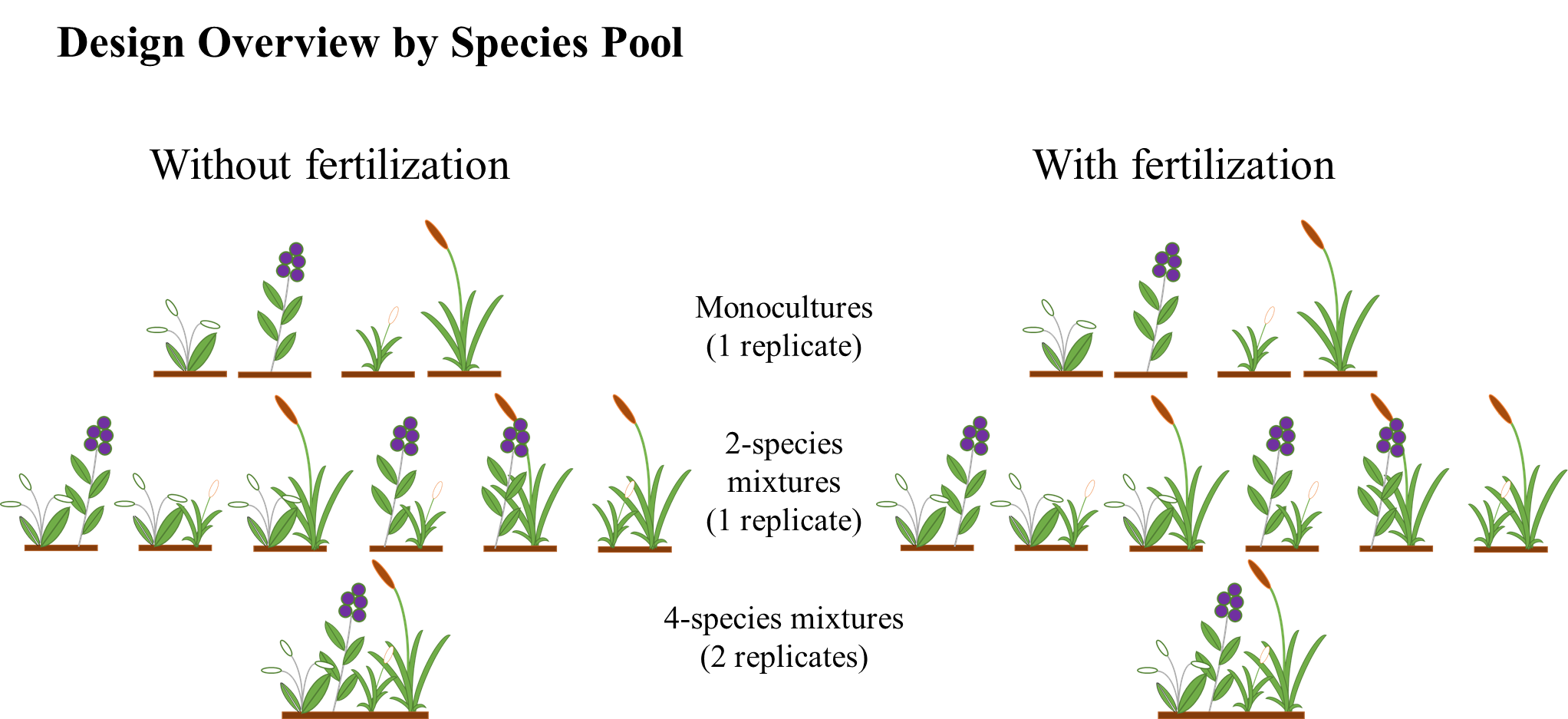


**Fig. S1** Overview of the experimental design of the Grassland Experiment (DivResource) for one species pool (= 24 plots × 2 species pools = 48 plots in total).


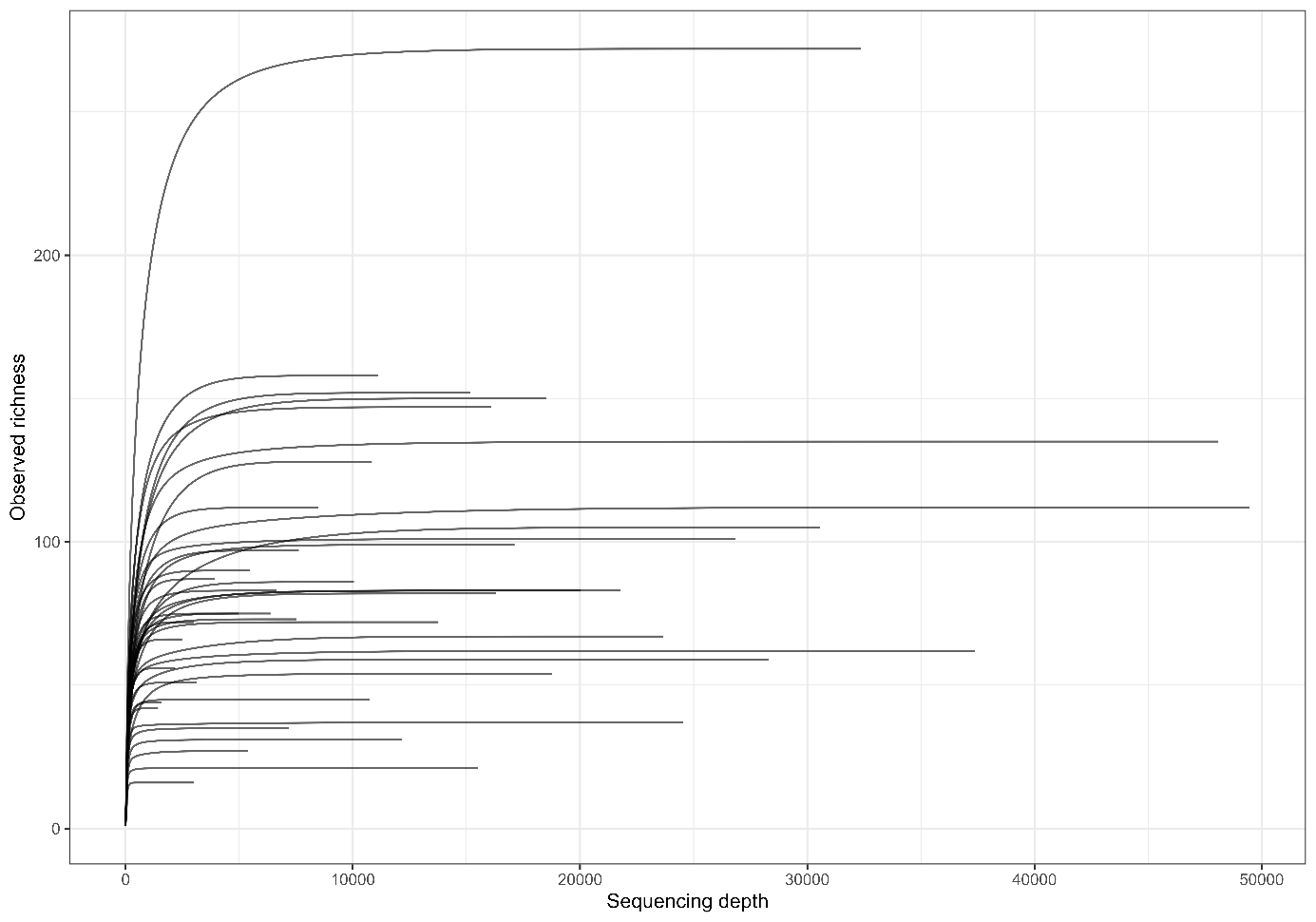


**Fig. S2** Rarefaction curves showing observed microeukaryotic richness as a function of sequencing depth across all samples.

**
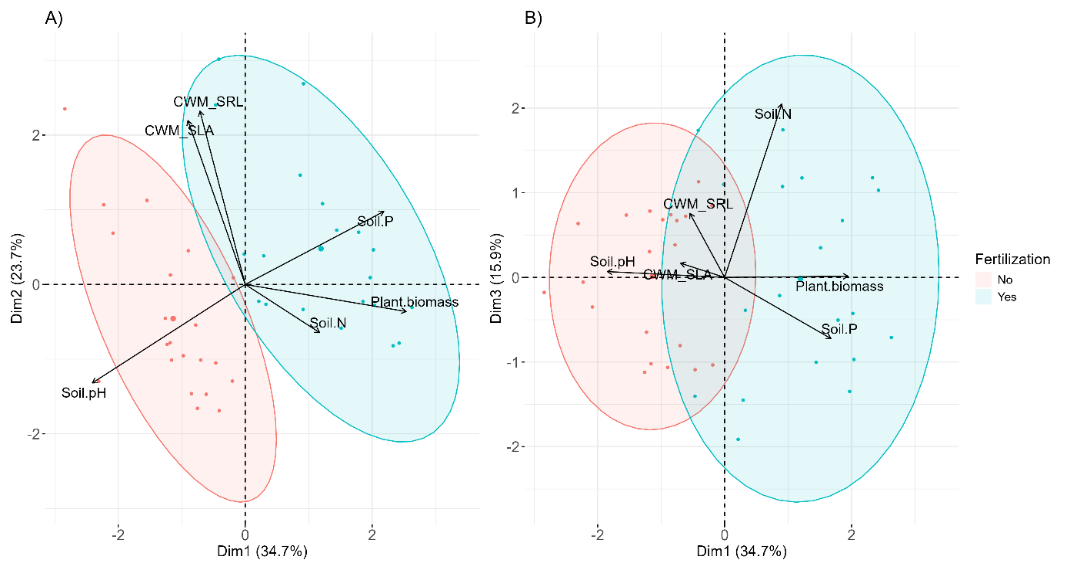
**

**Fig. S3** Multivariate ordination of soil properties, plant traits (SLA, SRL), and plant biomass in relation to fertilization treatment. (A) Principal component analysis (PCA) showing separation of samples along PC1 and PC2. (B) PCA showing separation along PC1 and PC3. Points represent individual plots, and coloured ellipses indicate 95% confidence intervals for fertilization treatments (red = no fertilization, blue = fertilization). Arrows represent environmental and plant trait vectors, with arrow length and direction indicating the strength and direction of correlations with ordination axes. Percentages on axes indicate the proportion of variance explained by each dimension.
